# Vascular Patterning affects Intramembranous Ossification through HIF1α-Vegf Signaling

**DOI:** 10.1101/2025.09.13.676037

**Published:** 2025-09-17

**Authors:** Soma Dash, Jonathan R. Rettig, Madelaine Gogol, Paul A. Trainor

**Affiliations:** 1 Department of Biological Sciences, University at Albany, SUNY Albany; 2 RNA Institute, University at Albany, SUNY Albany; 3 Stowers Institute for Medical Research; 4 University of Kansas, Medical Center

**Keywords:** Mediator complex, Vascular development, Craniofacial Development, Spatial transcriptomics

## Abstract

Organs and tissues develop in close association with vasculature which transports blood and nutrients and helps to remove waste. The vasculature is composed primarily of endothelial cells, which provide structure, form barriers, and are a source of developmental signals. We recently found that the Mediator, a multiprotein complex, which regulates transcription, was essential for proper vascular development. Here, we investigated the specific role of the Mediator tail subunit Med23 in endothelial cells. Endothelial specific knockout of *Med23* in mouse embryos using *Tek-Cre* results in vascular anomalies, including edema, hemorrhage, and mispatterned vasculature, alongside craniofacial defects such as micrognathia and cleft palate. Neural crest cell formation and migration were normal, however, osteogenic differentiation of neural crest cells was severely impaired in the craniofacial region in *Med23* mutants. Spatial transcriptomics revealed downregulated expression of key vascular and osteogenic genes, including *Vegfr1* and *Col1a1*, with altered signaling dynamics between endothelial and osteoblast populations. Elevated HIF1α expression and reduced VEGF signaling were observed in *Med23* mutants, suggesting a hypoxia-driven suppression of osteoblast maturation. Consistent with this model, pharmacological inhibition of HIF1α, combined with VEGFA supplementation, rescued craniofacial ossification and extended embryonic viability. These findings reveal a critical role for Med23 in coordinating vascular patterning and intramembranous ossification and highlight distinct hypoxic and angiogenic requirements in craniofacial dermal bone versus axial and appendicular endochondral bone development. Thus, the cranial vasculature and more specifically endothelial cells, play an instructive role in neural crest cell differentiation during craniofacial development.

## Introduction

Vertebrate bones originate from distinct embryonic sources depending on their anatomical location. For example, most of the craniofacial skeleton is derived from cranial neural crest cells and cranial mesoderm, whereas the axial skeleton is formed from the somites and the appendicular skeleton from lateral plate mesoderm^1^. While axial and appendicular bones typically form through endochondral ossification, involving the replacement of cartilage with bone, most craniofacial bones arise via intramembranous ossification, a distinct process in which cranial neural crest cell- or cranial mesoderm-derived mesenchyme directly differentiates into osteoblasts.

Bone development and repair are intimately linked to vascularization, with endothelial cells (ECs) providing angiocrine signals that support both osteogenesis and hematopoiesis^2,3^. Although the vascular dynamics of endochondral ossification are well characterized, the role of vasculature in intramembranous ossification remains poorly understood^4^. Notably, both ossification modes require avascular conditions at the initiation of primary ossification centers, followed by angiogenesis to sustain bone expansion^5^. This vascular influx not only supplies metabolic support but also ECs that interact with osteoblast precursors to regulate their differentiation and survival. ECs in the vasculature, depending on their proximity to the osteoblasts, have been shown to respond to pre-osteoblast cells, which then through various signaling pathways regulate the differentiation and survival of osteoblast cells^6,7^.

Osteoblasts and progenitors reciprocally influence EC behavior by secreting pro-angiogenic factors, including VEGF ligands. Hypoxia-induced VEGF signaling, mediated by HIF1α stabilization, is a key driver of both angiogenesis and osteogenesis^8–10^. HIF1α, a master regulator of the cellular response to hypoxia, promotes VEGF expression and enhances osteoblast differentiation in long bones under low oxygen conditions^11^. During development and regeneration through the endochondral ossification process, HIF1α is essential for coupling angiogenesis with osteogenesis^12^.

VEGF signaling plays a role in both angiogenesis and osteogenesis and its levels must be tightly regulated to ensure proper development^13^. Among the five VEGF ligands, Vegfa is the most abundant and functionally versatile, signaling primarily through Vegfr2 (Flk1, Kdr), while Vegfr1 (Flt1) acts as a decoy receptor reducing angiogenesis^14–16^. Regulation of VEGF signaling is tissue-specific and complex, involving transcriptional control by Runx2 and potential modulation by FGF and BMP pathways^17^.

Recent studies have highlighted the Mediator complex—a central integrator of transcriptional regulation—as a key player in craniofacial and vascular development^18–22^. The subunit Med23, in particular, has emerged as a critical regulator of endothelial cell function and cranial morphogenesis^18^. Conditional deletion of Med23 in endothelial cells leads to vascular defects including hemorrhage and diminished angiogenesis^20^. Loss of Med23 in neural crest cells results in craniofacial anomalies similar to Pierre-Robin sequence such as cleft palate and micrognathia, likely through perturbed modulation of WNT and Sox9 signaling pathways^19^. These findings underscore Med23’s role as a molecular bridge linking transcriptional control to morphogenetic and angiogenic processes during embryogenesis.

In this study, we describe an endothelial cell-specific knockout of Med23 in mice, which survive until embryonic day (E)16.5 and exhibit both vascular and craniofacial anomalies. The vascular defects, including edema and mispatterned vessels, arise from disruption of the HIF1α–VEGF signaling axis. Concurrently, we observe impaired craniofacial osteogenesis, marked by reduced expression of key osteogenic markers. Surprisingly, spatial transcriptomics reveal elevated expression of ossification-related genes in the mutants, particularly in proximity to vascular cells, suggesting precocious ossification in the jaw. This is accompanied by increased hypoxia near the mandibular bone. Consistent with these observations, pharmacological inhibition of HIF1α combined with VEGF pathway activation rescues the craniofacial ossification phenotype. Together, these findings support a model in which Med23 coordinates vascular and craniofacial development by regulating the spatial and temporal dynamics of ossification through vascular-mediated hypoxic signaling. Furthermore, our work reveals fundamental differences in the requirement and function for hypoxia and VEGF signaling during intramembranous ossification compared to endochondral ossification.

## Results

### Tissue specific deletion of Med23 from endothelial cells results in vascular anomalies

Development of the vertebrate head is a dynamic, complex process, which requires the coordinated integration of all three germ layers and their derivatives. In a forward genetic screen aimed at identifying novel regulators of craniofacial development^23^, we discovered a mouse mutant, *snouty,* which exhibited craniofacial and neurovascular anomalies but was early embryonically lethal^18,19^. We subsequently mapped the *snouty* mutation to a single nucleotide change in a ubiquitously expressed gene, *Med23*^18^, which encodes a subunit of the global transcription co-factor complex, the Mediator. To overcome the early embryonic lethality in *Med23*^*sn/sn*^ mutants and investigate the role of Med23 in later vascular development and maturation, we conditionally deleted *Med23* specifically in endothelial cells with *Tek-Cre* (*Tie2-Cre*) transgenic mice.

**Fig. 1:**
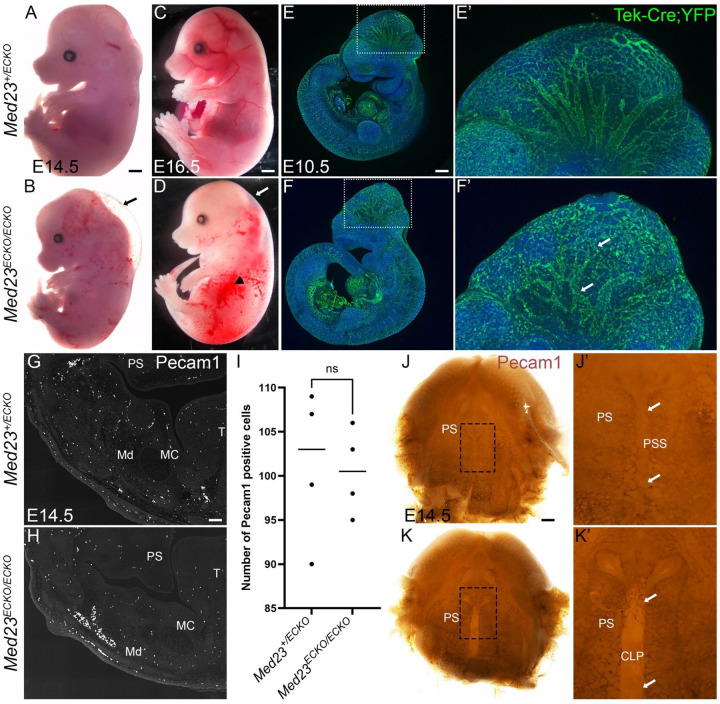
Vascular anomalies in *Med23*^*ECKO/ECKO*^ mutants. Bright field images of *Med23*^*+/ECKO*^ and *Med23*^*ECKO/ECKO*^ at E14.5 (A,B) and E16.5 (C,D) indicate that mutants exhibit vascular defects such as edema (indicated by arrows) and hemorrhage (arrowhead in D) (n=10). Lineage tracing using a Rosa-eYFP transgenic line followed by staining with GFP antibody indicates that the vascular patterning was disrupted in *Med23*^*ECKO/ECKO*^ compared to *Med23*^*+/ECKO*^ (n=5) (E,F). A high magnification image of the brain region is shown in E’ and F’. Arrows indicate the disrupted vasculature in *Med23*^*ECKO/ECKO*^ in F’. Pecam1 immunostaining on E14.5 sections indicates that the number of Pecam1 positive cells in the lower jaw is similar in *Med23*^*+/ECKO*^ and *Med23*^*ECKO/ECKO*^ (G,H) (n=4). However, the pattern of the Pecam1 positive cells, especially around the dentary bone (indicated by Md) is disrupted. Quantification of Pecam1 positive cells in the lower jaw is shown in I. Modified t-test was used for statistical analysis. Whole palatal shelf immunostained with antibody against Pecam1 indicates while the vasculature is not significantly reduced in the mutants, the patterning is altered (J,K) (n=6). (J’,K’) In the palatal shelf seam, there are new vascular cells in the anterior region and missing vasculature in the posterior region of the hard palate in the *Med23*^*+/ECKO*^ mutants compared to the *Med23*^*+/ECKO*^ controls (indicated by white arrows in J’, K’). Scale bar in A,B is 500 μm, C,D is 600 μm, E,F is 150 μm, G,H is 140 μm and J,K is 200 μm. Abbr. MC – Meckel’s Cartilage, Md – Mandible, T – Tongue, PS - Palatal Shelf, PSS – Palatal Shelf Seam, CLP – Cleft Palate

*Med23*^*fx/fx*^*;Tek-Cre* mutant embryos (hereby referred to as *Med23*^*ECKO/ECKO*^) survived until late gestation at E16.5 but presented with edema at E14.5 (Fig. 1A, B) that progressively worsened such that by E16.5, abdominal hemorrhage also became apparent (Fig. 1C, D). To investigate the developmental origin of these vascular anomalies, we performed lineage tracing of endothelial cell precursors using *Rosa-eYFP* reporter mice^24^.

While the vascular network was present in E10.5 *Med23*^*ECKO/ECKO*^ mutants, its patterning was notably altered particularly in the forebrain, midbrain, pharyngeal arches, and periocular regions compared to *Med23*^*+/ECKO*^ control embryos (Fig. 1E–F’). In contrast to the reiterated tree branch like pattern of large dorsoventrally oriented vessels connected to a well-organized polygonal network of smaller vessels in controls, the major vessels of the midbrain associated vascular plexus in *Med23*^*ECKO/ECKO*^ mutants were fewer, less directional, and connected to a disorganized network of micro vessels or capillaries, which was collectively indicative of altered vascular remodeling. The vascular plexus in the mandibular arch of *Med23*^*ECKO/ECKO*^ mutants appeared reduced compared to controls and this was further evident in sections of the mandible from the pattern of PECAM1 immunostaining of endothelial cells, especially around the dentary bone (indicated by Md) at E14.5. In the mandible, the number of Pecam1-positive endothelial cells appeared comparable between *Med23*^*+/ECKO*^ control and *Med23*^*ECKO/ECKO*^ mutant embryos (Fig. 1G, H). Quantification of PECAM1 positive cells in the lower jaw is shown in Figure 1I and a modified t-test was used for statistical analysis. PECAM1 immunostaining of whole palatal shelves revealed alterations in the pattern of the vasculature (Fig. 1J,K) with ectopic vasculature in the anterior palatal shelf seam (magnified image in Fig. 1J’, K’), and an absence of vasculature in the posterior region of the hard palate (indicated by white arrows) in *Med23*^*ECKO/ECKO*^ mutants compared to controls. However, we observed signs of neovascularization in the palatal seam of *Med23*^*ECKO/ECKO*^ mutants, which was suggestive of altered vascular remodeling.

### Tissue specific deletion of Med23 from endothelial cells results in craniofacial anomalies

**Fig. 2:**
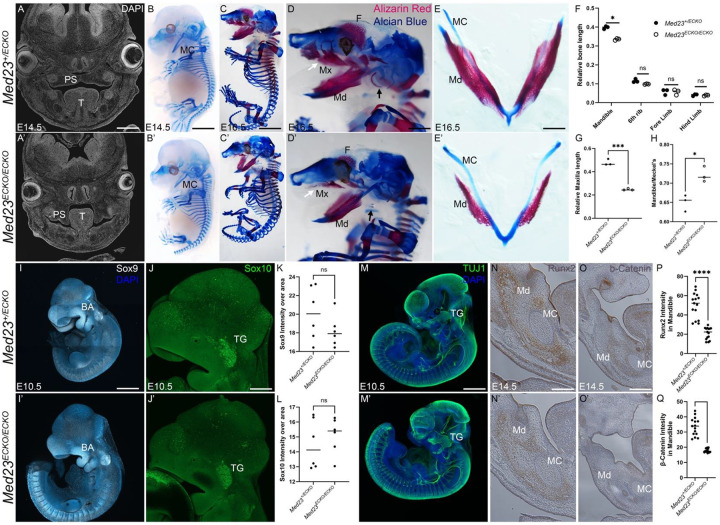
Craniofacial anomalies in *Med23*^*ECKO/ECKO*^ mutants. (A,A’) Sectioned and DAPI stained E14.5 embryos indicate that while the palatal shelf in the *Med23*^*+/ECKO*^ control embryos are fused in a horizontal position above the tongue, *Med23*^*ECKO/ECKO*^ mutants exhibit cleft palate with palatal shelves in a vertical position flanking the tongue (n=10). Scale bar = 150 μm. (B,B’) Alcian blue staining to reveal cartilage morphology indicates no substantial difference between *Med23*^*+/ECKO*^ and *Med23*^*ECKO/ECKO*^ at E14.5 (n=5). Scale bar = 200 μm. (C,C’) Alcian blue and alizarin red staining of E16.5 *Med23*^*+/ECKO*^ and *Med23*^*ECKO/ECKO*^ embryos indicates the presence of craniofacial osteogenesis defects (n=3). Scale bar = 300 μm. High magnification images of the craniofacial region are displayed in D and D’, which shows that the frontal bone, maxilla and mandible are drastically reduced in *Med23*^*ECKO/ECKO*^ embryos, while the tympanic bone is missing in the *Med23*^*ECKO/ECKO*^ embryos (indicated by black arrowheads). Scale bar = 400 μm. (E,E’) Reduced alizarin red staining in dissected lower jaw indicates that the mandible ossification is reduced in *Med23*^*ECKO/ECKO*^ embryos compared to *Med23*^*+ECKO*^. Scale bar = 75 μm. (F) Quantification of relative mineralized bone length compared to the body length (crown to rump) indicates that other than the mandible, no bones are altered significantly in size. Mandible length was normalized to the size of the head (nose to hind brain). (G) Quantification of the normalized size of the maxilla compared to the head size. (H) Quantification of the normalized size of the mandible compared to the Meckel’s cartilage. Immunostaining for neural crest cell markers, Sox9 (I,I’,K, scale bar = 250 μm) and Sox10 at E10.5 (J,J’,L, scale bar = 150 μm) suggests neural crest cells and neuronal cell progenitors are unaffected in the *Med23*^*ECKO/ECKO*^ embryos (n=6). (M,M’) Neuronal cells labeled by TUJ1 are similar between *Med23*^*+/ECKO*^ control and *Med23*^*ECKO/ECKO*^ mutant embryos (n=3). Scale bar = 250 μm. Osteoblast progenitors labeled by Runx2 (N,N’,P) and b-Catenin (O,O’,Q) indicate significantly reduced expression of both Runx2 and b-Catenin in the mandible (n = 5). Scale bar = 75 μm. Abbr. PS - Palatal Shelf, T – Tongue, MC - Meckel’s Cartilage, Md – Mandible, Mx- Maxilla, F- Frontal bone, BA- Branchial Arch, TG – Trigeminal Ganglion

Closer examination of *Med23*^*ECKO/ECKO*^ mutants revealed craniofacial anomalies, including micrognathia (Fig. 1B, D) and cleft palate (Fig. 2A, A’). To further characterize these defects, we performed skeletal staining with Alcian Blue and Alizarin Red to label cartilage and bone, respectively. At E14.5, craniofacial and axial cartilage development appeared normal in *Med23*^*ECKO/ECKO*^ mutants compared to *Med23*^*+/ECKO*^ controls, suggesting that Med23 in the endothelial cells is not essential for cranial chondrogenesis during embryogenesis (Fig. 2B, B’). However, by E16.5, while cartilage differentiation and growth remained unaffected, craniofacial osteogenesis was markedly reduced in the *Med23*^*ECKO/ECKO*^ mutant embryos, resulting in hypoplasia of the frontal, maxillary, tympanic, and mandibular bones (Fig. 2C – E’).

To assess the regional specificity of skeletal differentiation, we compared osteogenesis in the ribs and long bones to that in the craniofacial region. Notably, the mandible was significantly reduced in size, while other skeletal regions were unaffected (Fig. 2F). Maxillary ossification, normalized to the size of the head in the *Med23*^*ECKO/ECKO*^ mutants, was also significantly reduced compared to *Med23*^*+/ECKO*^ controls (Fig. 2G). Further analysis of the dentary bone revealed that mandibular length was significantly shorter in *Med23*^*ECKO/ECKO*^ mutants compared to *Med23*^*+/ECKO*^ controls when normalized to the length of Meckel’s cartilage (Fig. 2H). These findings suggest that vascular mispatterning resulting from *Med23* deletion in endothelial cells selectively impairs craniofacial osteogenesis, likely through disrupted signaling between the vasculature and osteoprogenitor cells.

### Neural crest cell migration is unaffected in the *Med23*^*ECKO/ECKO*^ mutants

Since most craniofacial bones are derived from neural crest cells (NCCs), we examined NCC migration at E10.5 using Sox9 and Sox10, which are also master regulators of cartilage and bone, and neuro-glial progenitors respectively^25–27^. Expression of each gene was comparable between *Med23*^*+/ECKO*^ control and *Med23*^*ECKO/ECKO*^ mutant embryos with Sox9 in the branchial arches and Sox10 in the trigeminal region, indicating that NCC induction and migration were not disrupted (Fig. 2I–L). The similar pattern of Sox9 expression also indicated that early chondrogenesis was unaffected, consistent with our Alcian blue skeletal staining results that showed cartilage development occurred normally in *Med23*^*ECKO/ECKO*^ mutant embryos. To assess cranial nerve development, we performed β-Tubulin III staining and observed intact and properly patterned cranial nerves and dorsal root ganglia in the mutants compared to controls (Fig. 2M–M’), demonstrating that NCC differentiation into neurons was unaffected by the alterations in the vasculature.

In contrast, markers of osteogenesis, including Runx2 and β-Catenin, were markedly reduced in the mandibular region of *Med23*^*ECKO/ECKO*^ mutants (Fig. 2N–Q), consistent with the decrease in Alizarin red staining. Runx2 is a master regulator of osteogenic differentiation^28^, while β-Catenin is a Wnt signaling effector molecule that is necessary for craniofacial bone formation^29^. These findings suggest that although NCCs successfully migrate to the mandibular arch, their differentiation into osteochondroprogenitors may be non-cell autonomously compromised by the absence of Med23 in endothelial cells, which implicates vascular signaling in the regulation of intramembranous bone ossification.

### Proliferation and Apoptosis Anomalies in *Med23*^*ECKO/ECKO*^ Mutants

**Fig. 3:**
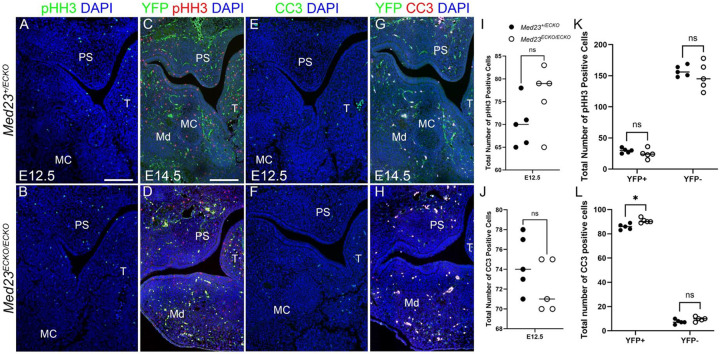
Proliferation and apoptosis anomalies of *Med23*^*ECKO/ECKO*^ mutants. Section immunostaining of *Med23*^*+/ECKO*^ controls and *Med23*^*ECKO/ECKO*^ mutants was performed with phospho-histone H3 (pHH3, green in A,B, red in C,D) and Cleaved Caspase 3 (CC3, green in E,F, red in G,H) at E12.5 and E14.5. E14.5 sections were also stained with YFP to lineage trace endothelial cells (C, D, G, H). n = 5. Scale bar is 70 μm. Quantifications of pHH3 and CC3 positive cells at E12.5 are shown in I and J, respectively. Quantifications of pHH3 and CC3 positive cells at E14.5 in the YFP+ (Endothelial cell) population vs YFP- cells are shown in K and L. Abbr. PS - Palatal Shelf, T – Tongue, MC - Meckel’s Cartilage, Md – Mandible

Considering craniofacial osteogenesis is markedly reduced in *Med23*^*ECKO/ECKO*^ mutants, we hypothesized that the osteochondroprogenitor population was reduced due to decreased proliferation or increased apoptosis. To assess cellular proliferation and apoptosis in *Med23*^*ECKO/ECKO*^ mutants, we performed immunostaining with antibodies against Phospho-Histone H3 (pHH3) and Cleaved Caspase-3 (CC3), respectively on coronal sections of E12.5 and E14.5 embryos. pHH3 staining revealed no changes in cell proliferation between *Med23*^*+/ECKO*^ control and *Med23*^*ECKO/ECKO*^ mutants E12.5 and E14.5 (Fig. 3A–D, I, K). Both endothelial derived tissues and surrounding mesenchymal cells have comparable numbers of pHH3 positive cells in E14.5 *Med23*^*+/ECKO*^ control and *Med23*^*ECKO/ECKO*^ mutant embryos. CC3 staining revealed a slight increase in apoptosis of vasculature associated endothelial cells in sections of E14.5 *Med23*^*ECKO/ECKO*^ mutants compared to *Med23*^*+/ECKO*^ controls, but not in the mandibular mesenchyme at either E12.5 or E14.5 (Fig. 3E–H, J, L).

These data suggest that loss of Med23 in endothelial cells disrupts vascular homeostasis by impairing endothelial cell survival, which may contribute to the vascular and craniofacial anomalies observed in *Med23*^*ECKO/ECKO*^ embryos.

### Spatial transcriptomics reveals transcriptomic changes in the osteochondroprogenitor cells

**Fig. 4:**
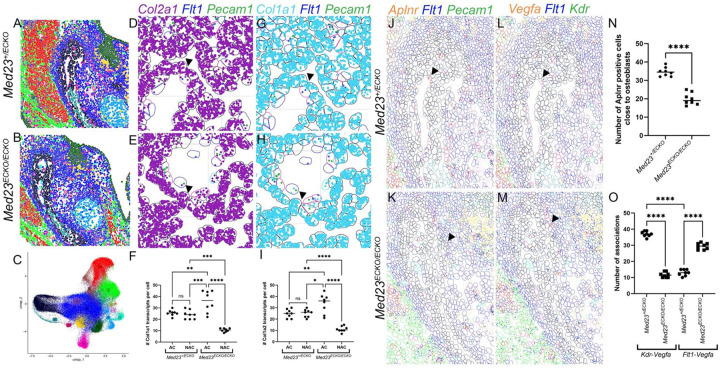
Spatial Transcriptomics indicates disruption of osteogenesis and angiogenesis genes. Spatial transcriptomics identifies tissue based on gene expression patterns of 990 genes that comprise a whole-body panel on the NanoString CosMx platform. (A-C) Uniform Manifold Approximation and Projection (UMAP) clustering and a feature plot of the major tissue types in the mandibular region led to the identification of 14 cell populations: Mesenchyme 1 (0, blue), Masseter Muscle (1, red), Mesenchyme 2 (2, light green), Immature Osteoblasts (3, black), Endothelial Cells (4, dark pink), Tooth (5, dark green), Dental Mesenchyme (6, yellow), Meckel’s Cartilage (7, light blue), Myogenic Progenitors (8, brown), Glia (9, teal), Neurons (10, purple), Mature Osteoblasts (11, dark teal), Neuron (12, light pink), Epithelial cells (13, olive green), and Pericytes (14, dark red). Spatial transcriptomics-based expression of *Col2a1* (purple, D-E) and *Col1a1* (cyan, G-H) in the adjoining (AC) osteoblasts (black outlined) compared to non-adjoining (NAC) endothelial cells (pink outlined) in *Med23*^*+/ECKO*^ controls and *Med23*^*ECKO/ECKO*^ mutants. Quantification of *Col2a1* transcripts (F) and *Col1a1* transcripts (I) were statistically analyzed using ANOVA. (J,K,N) Spatial transcriptomics-based expression of *Aplnr* (orange) in conjunction with *Flt1* (*Vegfr1*, blue) and *Pecam1* (green) around the mandible. (L,M) *Vegfa* (orange), *Flt1* (blue) and *Kdr* (*Vegfr2*, green) expression was analyzed and used to generate figures in R using Seurat. (O) Quantification of the number of *Vegfa* positive cells in proximity to *Vegfr1* positive cells compared to *Vegfa* positive cells in proximity to *Vegfr2* positive cells in *Med23*^*+/ECKO*^ controls and *Med23*^*ECKO/ECKO*^ mutants. Statistical analysis was performed using ANOVA.

To investigate the interactions between osteochondroprogenitor cells and the vasculature, we performed spatial transcriptomics using the CosMx platform (NanoString), which allowed us to profile 990 distinct transcripts across diverse cell types. We analyzed two coronal tissue sections each from two *Med23*^*+/ECKO*^ control and two *Med23*^*ECKO/ECKO*^ mutant embryos. Data were processed using the Seurat package in R and visualized via UMAP clustering to identify cell types based on gene expression, which were then mapped back to histological landmarks (Fig. 4A–C). This analysis revealed two distinct vascular cell populations, classified as endothelial cells and pericytes and two distinct osteoblast populations, classified as immature and mature osteoblasts.

Differential gene expression analysis (padj < 0.05, ≥20% expression in either group, and fold change >1.5) revealed three significantly altered genes in vascular cells: *Gnas, Aplnr,* and *Flt1* (Table 1). *Gnas* encodes the stimulatory G protein alpha subunit that regulates a variety of GPCR signaling. *GNAS* when mutated results in pseudopseudohypoparathyroidism which results in calcification of vasculature^30^. In addition, loss of function mutations in *GNAS* results in defects in cranial base as well as maxilla and mandible^31^. *Aplnr* encodes a G protein coupled receptor that binds Apelin, and Apelin signaling plays fundamental roles in vasculogenesis including the regulation of angiogenesis^32^. *Aplnr* expression was downregulated in *Med23*^*ECKO/ECKO*^ mutants, and disruption in Apelin signaling likely contributes to the vascular mispatterning observed in in the mutants (Fig. 4J, K, N). Flt1 (encoding VEGFR1) competes with VEGFR2 binding to VEGFA and in doing so plays important roles in modulating angiogenesis and vasculogenesis. *Flt1* was upregulated in *Med23*^*ECKO/ECKO*^ mutants, while *Vegfa* transcript levels remained unchanged (Fig. 4L, M). However, more endothelial cells expressed *Vegfa* in mutants, suggesting reduced per cell expression, which could indicate a response to disrupted vascular patterning and possible increased hypoxia.

With respect to osteoblasts, key matrix protein encoding genes which are also required for osteogenic differentiation including *Col1a1*, *Col1a2*, and *Sparc*^33,34^, were downregulated (Table 1) together with β-Catenin in mature osteoblasts^29^, which was confirmed by immunostaining (Fig. 2O, O’). Additionally, downregulation of *Thy1* and *Gja1* expression, which encode cell adhesion proteins that are important for craniofacial mesenchymal differentiation^35–37^, further indicate osteoblast maturation in the mutants is impaired—consistent with the skeletal phenotypes evident in *Med23*^*ECKO/ECKO*^ mutants (Table 1).

Spatial analysis revealed a striking pattern in *Med23*^*+/ECKO*^ control embryos, in which immature osteoblasts adjacent to and distant from vasculature expressed similar levels of osteogenic genes. In contrast, in *Med23*^*ECKO/ECKO*^ mutants, osteoblasts near vasculature exhibited elevated expression of *Col1a1* and *Col1a2*, while distant cells exhibited reduced expression of *Col1a1* and *Col1a2* (Fig. 4D–I). This is suggestive of enhanced osteogenesis in the vicinity of the vasculature in *Med23*^*ECKO/ECKO*^ mutants.

Interestingly, CellChat analysis of the spatial transcriptomics data revealed altered Vegf signaling dynamics (Fig. 4O, Table 2). Vegfa–Vegfr2 interactions were diminished in *Med23*^*ECKO/ECKO*^ mutants, while Vegfa–Vegfr1 interactions were enhanced compared to *Med23*^*+/ECKO*^ controls, supporting a shift in the balance of VEGF signaling that may underlie both vascular and osteogenic defects.

### Hypoxia is increased in *Med23*^*ECKO/ECKO*^ mutants

**Fig. 5:**
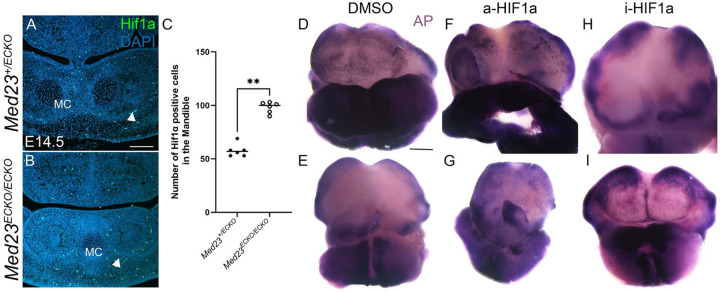
Hypoxia gene is upregulated in *Med23*^*ECKO/ECKO*^ mutants. (A,B) Coronal sections of E14.5 *Med23*^*+/ECKO*^ control and *Med23*^*ECKO/ECKO*^ mutant embryos were immunostained for HIF1α. Scale bar: 140 μm. (C) Quantification of HIF1α-positive cells in the mandible. Statistical analysis was performed using a non-parametric t-test; n = 6. (D–I) Ex-vivo mandible explants from *Med23*^*+/ECKO*^ controls and *Med23*^*ECKO/ECKO*^ mutants were treated with DMSO (D,E), HIF1α activator (a-HIF1α, F,G) and HIF1α inhibitor (i-HIF1α, H,I) followed by alkaline phosphatase staining to assess osteogenic activity. Scale bar: 500 μm.

VEGF signaling must be downregulated in long bones to promote osteoblast maturation and repair, a process mediated by hypoxia induced HIF1α signaling. We therefore investigated HIF1α signaling in *Med23*^*ECKO/ECKO*^ mutants and observed a significant upregulation of HIF1α expression, particularly around the dentary bone, which is suggestive of elevated hypoxia in *Med23*^*ECKO/ECKO*^ mutants compared to *Med23*^*+/ECKO*^ controls (Fig. 5A–C).

However, this observation contrasts with established models of endochondral ossification, where increased HIF1α and reduced VEGF signaling promote osteoblast differentiation^11,38^. Despite similar molecular changes, mandibular osteogenesis is markedly reduced in *Med23*^*ECKO/ECKO*^ mutants compared to *Med23*^*+/ECKO*^ control embryos. This discrepancy raises the possibility that intramembranous ossification may have distinct vascular and hypoxic requirements compared to endochondral ossification.

To analyze the effect of HIF1α upregulation in *Med23*^*ECKO/ECKO*^ mutants, we performed ex-vivo mandibular explant cultures in the presence of a HIF1α activator and inhibitor. When treated with the HIF1α activator, DMOG, *Med23*^*ECKO/ECKO*^ mutant explants did not exhibit altered alkaline phosphatase staining in comparison to *Med23*^*+/ECKO*^ control vehicle treated explants (Fig. 5D–G), suggesting no effect on the state of osteogenic differentiation. In contrast, when mandibular explants were treated with the HIF1α inhibitor, FM19G11, both *Med23*^*+/ECKO*^ control and *Med23*^*ECKO/ECKO*^ mutant explants exhibited increased alkaline phosphatase staining in comparison to *Med23*^*+/ECKO*^ and *Med23*^*ECKO/ECKO*^ mutant treated with DMSO, consistent with enhanced osteogenesis (Fig. 5D, E, H and I).

### VEGF-HIF1α modulation rescues osteogenesis in *Med23*^*ECKO/ECKO*^ mutants

**Fig. 6:**
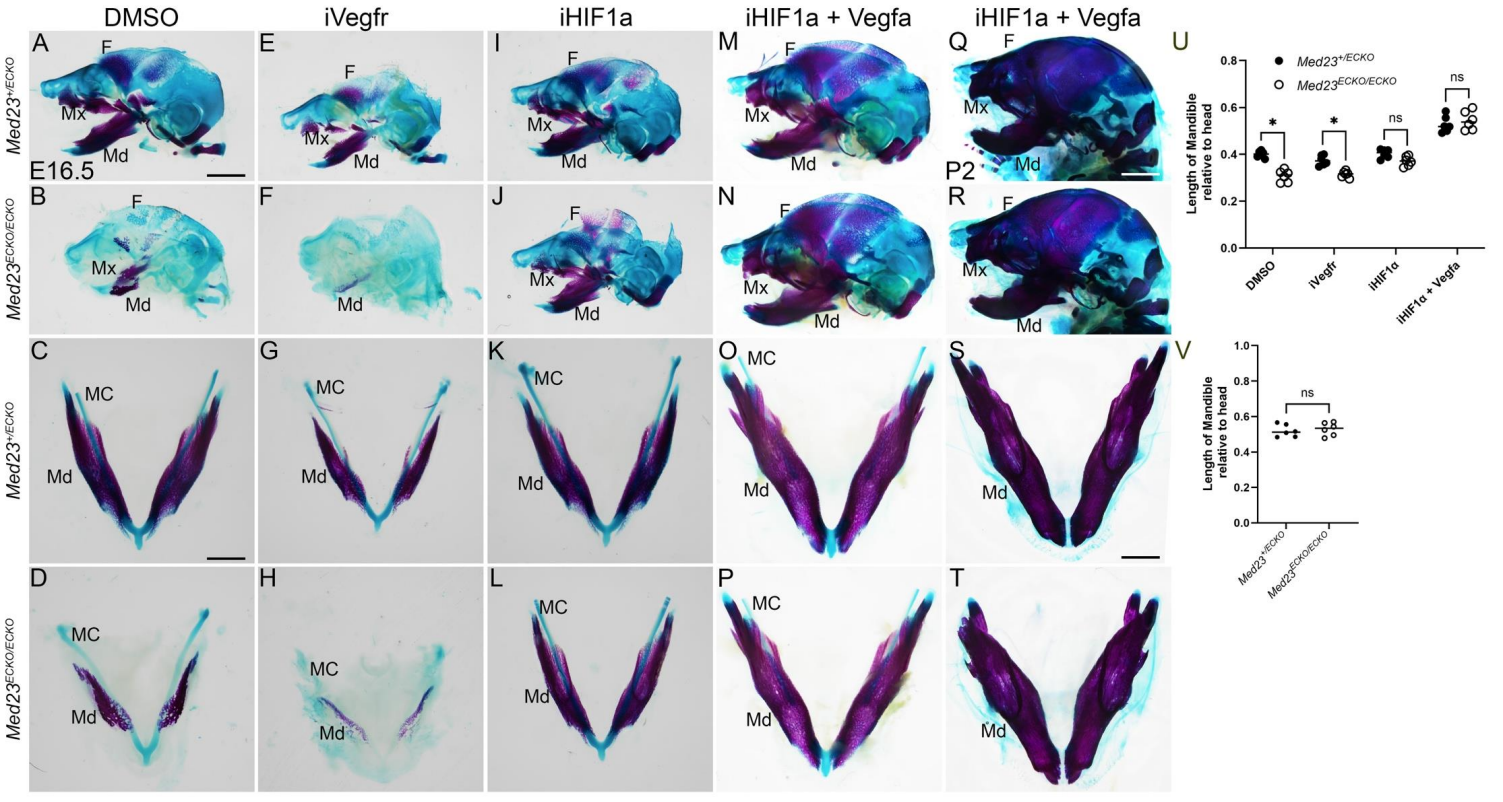
Osteogenesis rescue in *Med23*^*ECKO/ECKO*^ mutants with Vegf-HIF1a modulation. Alcian blue and alizarin red staining of *Med23*^*+/ECKO*^ controls and *Med23*^*ECKO/ECKO*^ mutants treated with DMSO (A-D), Vegfr inhibitor (iVegfr, E-H), Hif1a inhibitor (iHIF1a, I-L) and Hif1a inhibitor combined with Vegfa (iHIF1a + Vegfa, M-P) at E16.5 reveals partial rescue of craniofacial osteogenesis using iHIF1a and complete rescue with iHIF1a + Vegfa. (Q-R) Embryos treated with iHIF1a + Vegfa survive until P2 unlike DMSO treated embryos. At P2, the mandible size along with other craniofacial bones are comparable between *Med23*^*+/ECKO*^ controls and *Med23*^*ECKO/ECKO*^ mutants indicating complete rescue of the mutants. (U) Quantification of length of the mandible relative to the length of the head (n=6) of E16.5 embryos treated with DMSO, iVegfr, iHIF1a and iHIF1a + Vegfa. Mann-Whitney U test was performed for statistical analysis. (V) Quantification of the length of the mandible relative to the length of the head (n=6) treated with iHIF1a + Vegfa of both *Med23*^*+/ECKO*^ controls and *Med23*^*ECKO/ECKO*^ mutants at P2. Scale bar for A,B, E, F, I, J, M, N is 200 μm. Scale bar for C, D, G, H, K, L, O, P is 75 μm. Scale bar for Q, R is 350 μm. Scale bar for S, T is 85 μm. Abbr. MC - Meckel’s Cartilage, Md – Mandible, Mx- Maxilla, F- Frontal bone

Our data collectively suggested that elevated HIF1α and altered VEGF signaling perturbed craniofacial bone formation in *Med23*^*ECKO/ECKO*^ mutants. To test this idea, we performed pharmacological inhibition and activation of VEGF signaling in combination with inhibition of HIF1α signaling to investigate the mechanistic effects of VEGF-HIF1α signaling on intramembranous ossification. To assess the role of VEGF signaling on craniofacial bone formation in *Med23*^*ECKO/ECKO*^ mutants, we treated pregnant dams once at E9.5 and again at E13.5 with the VEGF inhibitor ZM323881. As expected, VEGF signaling inhibition disrupted osteogenesis in *Med23*^*+/ECKO*^ control embryos compared to DMSO carrier controls. However, *Med23*^*ECKO/ECKO*^ mutant embryos exhibited minimal additional disruption, likely due to the fact that that VEGF signaling is already significantly reduced in *Med23*^*ECKO/ECKO*^ mutants such that further VEGF signaling suppression does not exacerbate the phenotype (Fig. 6A–H).

In contrast, *Med23*^*+/ECKO*^ embryos treated with the HIF1α inhibitor FM19G11, exhibited no change in osteogenesis as measured by Alizarin Red staining and length of the mandible. However, *Med23*^*ECKO/ECKO*^ mutants subjected to HIF1α inhibition exhibited a marked increase in osteogenesis, particularly in the mandibular and maxillary regions (Fig. 6I–L). This result implicates HIF1α-mediated signaling as a key contributor to the impaired ossification observed in *Med23*^*ECKO/ECKO*^ mutants.

The decreased VEGF signaling and increased HIF1α signaling observed in *Med23*^*ECKO/ECKO*^ mutant embryos led us to hypothesize that combinatorial inhibition of HIF1α signaling together with restoration of VEGF signaling could rescue the osteogenic anomalies. We therefore treated pregnant dams with the HIF1α inhibitor, FM19G11 and purified VEGFA at E9.5 and E13.5 and observed a substantial rescue of the craniofacial ossification phenotype in *Med23*^*ECKO/ECKO*^ mutants, as evidenced by restoration of bone formation in the mandible, maxilla, and frontal bones (Fig. 6M–P). We quantified mandible lengths relative to the length of the head (tip of snout to hind brain) and observed that mandible lengths of *Med23*^*+/ECKO*^ control and *Med23*^*ECKO/ECKO*^ mutants treated with a combination of HIF1α inhibitor and VEGFA are comparable to each other (Fig. 6U).

Furthermore, whereas *Med23*^*ECKO/ECKO*^ mutants are typically embryonic lethal by E16.5, combined HIF1α inhibition and VEGFA supplementation prolonged their lifespan for a limited period postnatally. Postnatal day (P) 2 *Med23*^*ECKO/ECKO*^ mutants exhibited no gross structural craniofacial bone defects and were indistinguishable from *Med23*^*+/ECKO*^ controls (Fig. 6Q–T, V). However, we were unable to collect pups *Med23*^*ECKO/ECKO*^ mutants at P7, for reasons which remain to be determined.

**Fig. 7:**
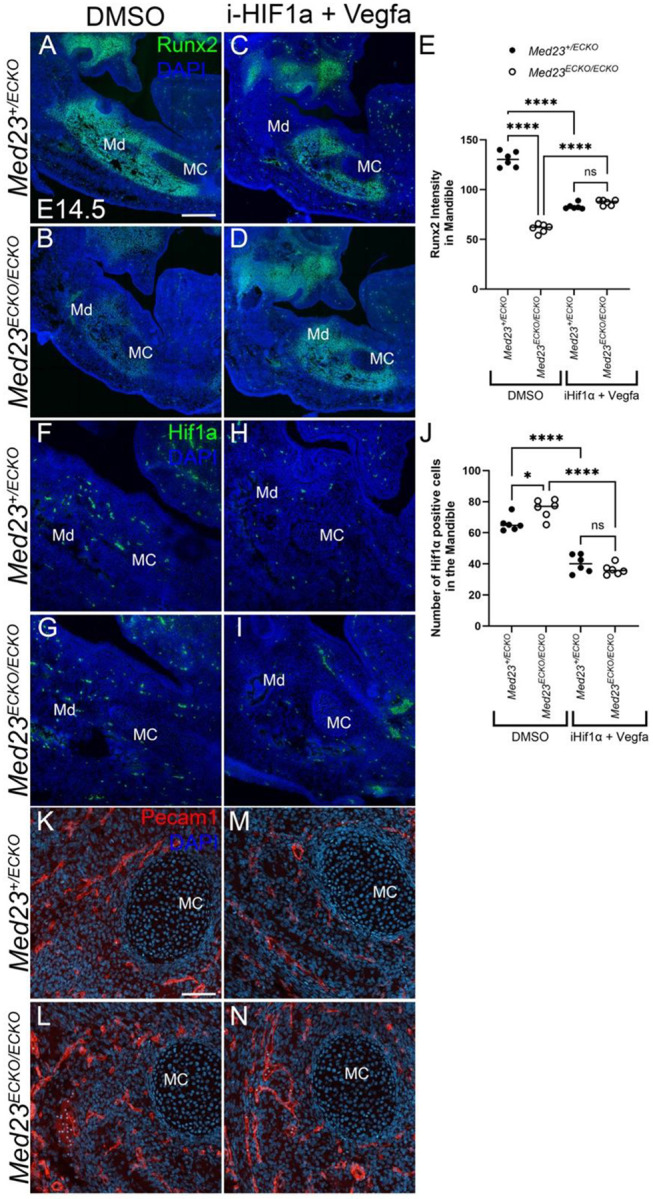
Osteogenesis and angiogenesis rescue of *Med23*^*ECKO/ECKO*^ mutants. *Med23*^*+/ECKO*^ control and *Med23*^*ECKO/ECKO*^ mutant embryos were treated with either DMSO (A, B, F, G, K, L) or a combination of HIF1α inhibitor and recombinant VEGFA (i-HIF1α + Vegfa; C, D, H, I, M, N). Immunostaining was performed for Runx2 (green; A–D), HIF1α (green; F–I), and Pecam1 (red; K–N). Scale bars: 280 μm for panels A–I; 70 μm for panels K–N. (E) Quantification of Runx2 intensity in the mandible (intensity/area). (J) Quantification of HIF1α-positive cells in the mandible. Statistical analysis was performed using one-way ANOVA followed by multiple t-tests. Abbr. MC - Meckel’s Cartilage, Md – Mandible.

To define the mechanisms underlying the rescue of vascular and osteogenic development, we performed Runx2, HIF1α and Pecam1 staining on DMSO control and HIF1α inhibitor and VEGFA treated *Med23*^*+/ECKO*^ control and *Med23*^*ECKO/ECKO*^ mutant embryos at E14.5. Runx2 staining revealed restored expression in concert with normal bone size in HIF1α inhibitor and VEGFA treated *Med23*^*ECKO/ECKO*^ mutants comparable to treated *Med23*^*+/ECKO*^ controls, consistent with the rescue of osteogenesis (Fig. 7A–E). The number of HIF1α positive cells was also reduced in both *Med23*^*+/ECKO*^ control and *Med23*^*ECKO/ECKO*^ mutant embryos treated with HIF1α inhibitor and VEGFA compared to DMSO treated embryos as would be expected from HIF1α inhibition (Fig. 7F–J). PECAM1 staining in the mandible revealed comparable number and distribution of endothelial cells in *Med23*^*+/ECKO*^ control and *Med23*^*ECKO/ECKO*^ mutant embryos treated with HIF1α inhibitor and VEGFA (Fig. 7K–N).

**Fig. 8:**
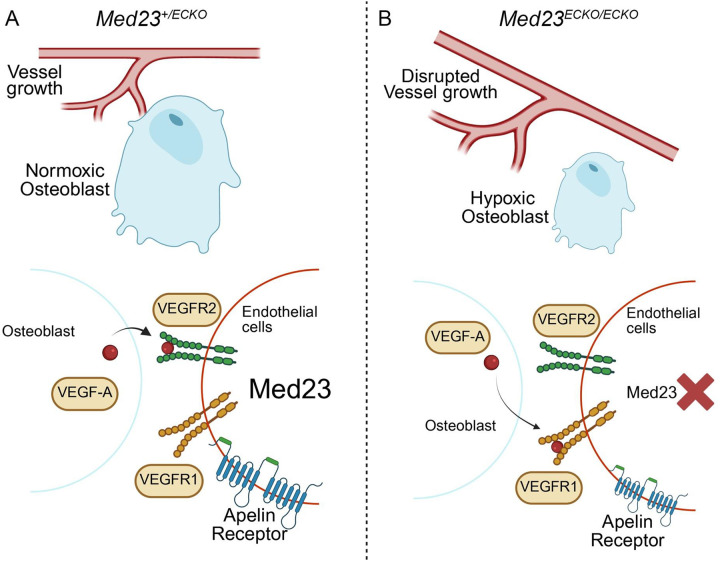
Decreased VEGF signaling combined with increased hypoxia disrupts intramembranous ossification. (A) In wild-type craniofacial tissue, endothelial cells expressing Med23 maintain Apelin receptor (Aplnr) expression, which guides vascular branching within the cranial mesenchyme. VEGFA secreted by nearby pre-osteoblasts binds to VEGFR2 on endothelial cells, promoting both angiogenesis and osteogenesis. (B) In *Med23*^*ECKO/ECKO*^ mutants, Aplnr expression is reduced, leading to aberrant vascular patterning in the mesenchyme of both the upper and lower jaw. VEGFA signaling is disrupted as it preferentially binds to VEGFR1, a decoy receptor, rather than VEGFR2, resulting in diminished osteogenic support and impaired craniofacial bone formation.

Together, these findings demonstrate that vascular patterning critically influences craniofacial bone development. Upregulation of HIF1α in *Med23*^*ECKO/ECKO*^ mutants leads to reduced VEGF signaling and impaired ossification, which can be reversed through targeted modulation of HIF1α and VEGF signaling (Fig. 8).

## Discussion

In this study, we showed that endothelial cell deletion of Med23 results in mid-gestation lethality around E16.5 in association with edema, hemorrhage, and craniofacial anomalies. Interestingly, a previous *Med23* loss-of-function study, also using *Tek-Cre,* resulted in lethality at E12.5^20^, in association with similar cranial vascular defects, edema and hemorrhage. Collectively these studies illustrate that Med23 plays an important role in vascular development. However, in addition to a critical role for endothelial-specific Med23 in coordinating vascular patterning, our study has uncovered a non-cell autonomous effect on craniofacial bone development during embryogenesis. While previous work has implicated Med23 in transcriptional regulation and morphogenesis, our findings extend its function to the regulation of intramembranous ossification via vascular mediated signaling.

NCCs, the progenitors of most craniofacial bone are known to migrate along vascular networks during embryogenesis, using endothelial structures as guidance cues to reach their target destinations^39^. Despite an abnormal vascular network, NCC migration and differentiation into neurons and chondrocytes in *Med23*^*ECKO/ECKO*^ embryos was normal, as was confirmed by Sox9 and Sox10 expression and cranial nerve development. However, neural crest cell derived osteogenesis in the craniofacial region was severely impaired.

This dissociation between NCC migration and bone formation suggests that endothelial cell-derived signals are critical for the differentiation of post-migratory NCCs into osteochondroprogenitor and osteoblast lineages during craniofacial bone development. Recent studies have shown that endothelial cells regulate osteogenesis through paracrine signaling, including VEGF and Notch pathways, which are essential for osteoblast maturation and bone matrix deposition^40^. In *Med23*^*ECKO/ECKO*^ mutants, disruption of vascular patterning and altered HIF1α signaling likely interfere with these endothelial-osteoblast interactions, leading to reduced expression of key osteogenic markers, such as Runx2 and β-Catenin. These findings support a model in which Med23 in endothelial cells is cell autonomously required not only for vascular integrity but also non-cell autonomously for the proper signaling environment that enables NCC-derived osteoprogenitors to differentiate and form craniofacial bone.

The VEGF signaling pathway plays a central role in mediating bone–vascular interactions during development and regeneration. Knockout of Vegfr1 leads to excessive angiogenesis and embryonic lethality, underscoring its critical role in maintaining vascular balance during development^41^. VEGF signaling has been shown to stabilize β-Catenin activity, a key regulator of osteoblast differentiation, particularly during endochondral ossification^42^. Our findings provide a corollary to this mechanism in the context of intramembranous ossification. In *Med23*^*ECKO/ECKO*^ mutants, we observe a reduction in VEGF signaling, which coincides with diminished β-Catenin expression in the mandibular bone. Given the distinct developmental origins and ossification mechanisms of craniofacial versus long bones, our data support the hypothesis that intramembranous ossification may have unique requirements for VEGF signaling and its downstream effectors.

Moreover, VEGF signaling itself influences β-Catenin stability, which is crucial for osteoblast maturation. Studies have shown that VEGF can stabilize β-Catenin during endochondral bone development, promoting osteogenic differentiation^42^. In contrast, reduced VEGF signaling—whether due to genetic disruption or altered hypoxic signaling—can lead to impaired β-Catenin activity and diminished bone formation.

In bone development, particularly during endochondral ossification, VEGF signaling is regulated by HIF1α to facilitate vascular invasion into a cartilage template, which is essential for osteoblast recruitment and bone matrix deposition^43^. However, the relationship between HIF1α-VEGF signaling and osteogenesis is nuanced. While HIF1α promotes Vegfa transcription^44^ and osteogenesis, excessive HIF1α activity can inhibit osteoblast differentiation by upregulating Twist2, which suppresses Runx2^45^. Overexpression of HIF1α in osteoblasts has also been shown to increase bone mass and vascular density^46^, while its inhibition impairs bone formation and vascular invasion^47,48^. Interestingly, HIF1α exhibits age-dependent effects: in young bone, it promotes osteogenesis and angiogenesis, whereas in aged bone, elevated HIF1α correlates with impaired bone-vascular coupling, potentially through a ROS-mediated p53 pathway^12^. This dual role suggests that HIF1α must be tightly regulated to balance angiogenesis and osteogenesis.

Interestingly, our data showed that HIF1α expression was elevated and VEGF signaling was reduced in *Med23*^*ECKO/ECKO*^ mutants, particularly near the mandibular bone. This pattern mirrors the signaling dynamics observed during endochondral ossification, where hypoxia-induced HIF1α promotes osteoblast differentiation through VEGF suppression^6^. However, in the context of intramembranous ossification, these same molecular cues appear to inhibit bone formation, suggesting a fundamental difference in the vascular dependency of these two ossification processes.

Pharmacological manipulation of hypoxia and VEGF signaling further supports this distinction. Treatment with a VEGF inhibitor disrupted osteogenesis in controls but had minimal effect on *Med23*^*ECKO/ECKO*^ mutants, consistent with already reduced VEGF signaling. Conversely, inhibition of HIF1α rescued craniofacial bone formation in the mutants, and combined treatment with a HIF1α inhibitor and exogenous VEGFA fully restored ossification and extended embryonic viability in *Med23*^*ECKO/ECKO*^ mutants. These findings underscore the importance of tightly regulated hypoxic and angiogenic signaling in craniofacial development and highlight Med23 as a key molecular integrator of these pathways.

In summary, our data demonstrate that Med23 is essential for proper vascular patterning and craniofacial ossification, acting through modulation of HIF1α-VEGF signaling. The differential response of intramembranous versus endochondral ossification to hypoxia and VEGF underscores the need to consider tissue-specific vascular requirements in skeletal development and disease and has the potential to improve bone graft repair of critical sized intramembranous craniofacial bone defects. Future studies will be needed to dissect the downstream transcriptional networks regulated by Med23 and to explore its potential as a therapeutic target in congenital craniofacial disorders.

## Methods

### Mouse husbandry

Mice were maintained under standard housing conditions following approved IACUC protocol 23–013 at the University at Albany, SUNY Albany and 2025–184 at the Stowers Institute for Medical Research. *Med23flox* animals were obtained from KOMP and maintained as previously described^18^. Similarly, *Tek-Cre* (Tie2-Cre) (B6.Cg-Tg(Tek-Cre)1Ywa/J) and *Rosa-eYFP* (B6.129X1-*Gt(ROSA)26Sor*^*tm1(EYFP)Cos*^/J) mice were obtained from JAX and maintained as previously described^18,19^. For timed matings, the day a vaginal plug was found was designated as embryonic day 0.5 (E0.5). *Med23*^*fx/fx*^*;Tek-Cre* (*Med23*^*ECKO/ECKO*^) and *Med23*^*+/fx*^*;Tek-Cre* (*Med23*^*+/ECKO*^) embryos were used as mutants and controls for every experiment, respectively.

### Brightfield imaging and Skeletal staining

Embryos were dissected at the desired stages in PBS, anesthetized in ice cold PBS for an hour and imaged using a Nikon DS-Ri camera or a Nikon DS10 Digital Camera. For skeletal preparation and staining, embryos were collected and fixed in 95% ethanol, followed by Alcian blue and Alizarin red staining of cartilage and bone as previously described^49^. Stained embryos were imaged using a Nikon DS-Ri camera or a Nikon DS10 Digital Camera. For quantification, bone length was measured in ImageJ.

### Lineage tracing

*Med23*^*fx/+*^*;Tek-Cre* mice were crossed with *Rosa-eYFP* mice to generate *Med23*^*fx/+*^*;Tek-Cre;YFP+* and *Med23*^*fx/fx*^*;Tek-Cre;YFP+* embryos. These animals were then immunostained with a GFP antibody as described below.

### Immunostaining

Whole embryo and dissected palates were fixed in 4% paraformaldehyde overnight followed by dehydration through an ascending methanol series into 100% methanol, after which they were stored at −20C until further use as described previously^19^. The tissues were then treated with Dent’s bleach (4:1:1 Methanol : DMSO : Hydrogen peroxide), rehydrated, and processed for immunostaining as previously described^19,50^.

Tissue sections both FFPE and frozen were stained according to standard protocols. Antibodies used were: Pecam1 (CD31, 1:100, BD Pharmingen #553370), Cleaved Caspase 3 (1:100, Cell signaling #9661S), Phospho-Histone H3 (1:1000, Millipore #06–570), Sox9 (1:500, Abcam #ab185966), Sox10 (1:500, Abcam #ab155279), Tuj1(1:1000, Biolegend #657402), Runx2 (1:500, Abcam #ab192256), b-Catenin (1:500, Abcam #ab32572) and HIF1α (1:1000, Abcam #ab228649). Each experiment consisted of 5 biological replicates and 3 technical replicates. ImageJ was used for analysis and quantification. For quantification, technical replicates were averaged per sample. Intensity measurements were normalized to the area.

### Spatial Transcriptomics

E14.5 *Med23*^*+/ECKO*^ and *Med23*^*ECKO/ECKO*^ embryos were dissected in PBS, fixed in 4% paraformaldehyde and then embedded in paraffin. The samples were sectioned serially at 10um. One of the slides with serial sections was stained with Hematoxylin and Eosin, whereas other slides were used for spatial transcriptomics with the NanoString platform. The experiment was performed using two technical replicates and two biological replicates. Analysis of Spatial Transcriptomics was performed using Seurat Package (4.9.9.9041) in R (4.2.0).

### Ex-vivo Mandible culture

The mandibular portion of branchial arch 1 was dissected from E11.5 *Med23*^*+/ECKO*^ and *Med23*^*ECKO/ECKO*^ embryos and cultured in equilibrated complete media on cell inserts in a 37C incubator in 5% Carbon dioxide for 72 hours with media change every 24 hours. The complete media contains BGJb media, 20% FBS, 1 mg/ml L-Glutamine, 0.1 mg/ml Vitamin-C, 1% 2-mercaptoethanol and 1% Penicillin-Streptomycin. Drug treatment began after 4 hours of equilibration in the complete media. Drugs used included HIF1α inhibitor, FM19G11 (5mg/kg, Selleckchem #E1712), HIF1α activator, DMOG (3mg/kg, Selleckchem #S7483). A total of 5 *Med23*^*+/ECKO*^ and 5 *Med23*^*ECKO/ECKO*^ embryos were used for the experiment.

### Drug treatments for rescues

Pregnant dams were treated with the drugs mentioned below via intraperitoneal injection once at E9.5 and again at E13.5. Drugs included HIF1α inhibitor, FM19G11 (20mg/kg, Selleckchem #E1712), HIF1α activator, DMOG (10mg/kg, Selleckchem #S7483), VEGF Signaling inhibitor, ZM323881 (10mg/kg, Selleckchem # S2896) and VEGF signaling activator, VEGFA (50ug/kg, Abnova, # P4608). DMSO was used as a vehicle control. The embryos were collected at E14.5 and E16.5 and neonatal pups were collected at P2 and P7 for end point experiments.

### Statistical tests

Student t-tests and ANOVA with multiple t-tests were performed based on the number of numerical variables and comparisons. When applicable non-parametric Mann-Whitney U-tests were performed for multiple comparisons. P value of <0.05 was designated with one asterisk *, <0.005 with **, <0.0001 with *** and <0.00001 with ****.
